# Accounting for Population Structure in Gene-by-Environment Interactions in Genome-Wide Association Studies Using Mixed Models

**DOI:** 10.1371/journal.pgen.1005849

**Published:** 2016-03-04

**Authors:** Jae Hoon Sul, Michael Bilow, Wen-Yun Yang, Emrah Kostem, Nick Furlotte, Dan He, Eleazar Eskin

**Affiliations:** 1 Department of Psychiatry and Biobehavioral Sciences, University of California, Los Angeles, Los Angeles, California, United States of America; 2 Computer Science Department, University of California, Los Angeles, Los Angeles, California, United States of America; 3 Department of Human Genetics, University of California, Los Angeles, Los Angeles, California, United States of America; University of California San Diego and The Scripps Research Institute, UNITED STATES

## Abstract

Although genome-wide association studies (GWASs) have discovered numerous novel genetic variants associated with many complex traits and diseases, those genetic variants typically explain only a small fraction of phenotypic variance. Factors that account for phenotypic variance include environmental factors and gene-by-environment interactions (GEIs). Recently, several studies have conducted genome-wide gene-by-environment association analyses and demonstrated important roles of GEIs in complex traits. One of the main challenges in these association studies is to control effects of population structure that may cause spurious associations. Many studies have analyzed how population structure influences statistics of genetic variants and developed several statistical approaches to correct for population structure. However, the impact of population structure on GEI statistics in GWASs has not been extensively studied and nor have there been methods designed to correct for population structure on GEI statistics. In this paper, we show both analytically and empirically that population structure may cause spurious GEIs and use both simulation and two GWAS datasets to support our finding. We propose a statistical approach based on mixed models to account for population structure on GEI statistics. We find that our approach effectively controls population structure on statistics for GEIs as well as for genetic variants.

## Author Summary

Although genome-wide association studies (GWASs) have discovered numerous novel genetic variants associated with many complex traits and diseases, those genetic variants typically explain only a small fraction of phenotypic variance. Factors that account for phenotypic variance include environmental factors and gene-by-environment interactions (GEIs). Recently, several studies have conducted genome-wide gene-by-environment association analyses and demonstrated important roles of GEIs in complex traits. One of the main challenges in these association studies is to control effects of population structure that may cause spurious associations. In this paper, we show both analytically and empirically that population structure may cause spurious GEIs and use both simulation and two GWAS datasets to support our finding. We propose a statistical approach based on mixed models that can effectively correct for population structure when searching for GEIs.

## Introduction

Over the past decade, genome-wide association studies (GWASs) have been a predominant approach to identify genetic variants involved in many complex traits and diseases.[1–3] While GWASs have discovered associations of many genetic variants, a large proportion of phenotypic variance for most traits is not explained by these variants.[4] Among several possible factors that explain this phenotypic variance such as effects of rare variants and epistasis, gene-by-environment interactions (GEIs) have drawn significant attention because of their important effect in many traits and diseases.[5–8] Discovering GEIs involved in diseases is of major interest in genetic research because they can provide insight into disease pathways, an understanding of the effect of environmental factors in disease, better risk prediction and personalized therapies. Similar to traditional GWASs that attempt to detect associations of genetic variants, researchers have recently performed gene-by-environment genome-wide association studies (GxE GWASs) to identify GEIs associated with diseases.[9–11]

One major difficulty in association studies is that population structure can easily confound the studies.[12] Association studies assume that individuals are unrelated, and if they are not, inflation of test statistics and possibly spurious associations may arise if genetic relatedness within individuals is imprecisely modeled. Several statistical approaches have been proposed to address this problem including genomic control [13], principal components analysis [14], and linear mixed models.[15] In particular, methods based on linear mixed models which incorporate pairwise relatedness between individuals has been shown to capture complex sample structure more effectively than other methods.[15, 16] It is important to note that all of these methods are designed to correct for population structure on statistics for genetic variants.

In contrast to numerous studies that have analyzed effect of population structure on association statistics in real GWAS datasets, few studies have investigated its effect on GEI statistics empirically. There have been, however, a few studies that evaluated bias caused by population structure on GEI statistics through simulations. Wang et al.[17] showed that population structure may have small effect on GEI statistics when genetic variants and environments have small correlations while Cheng and Lee [18] showed that it may introduce unacceptable bias to the estimation of GEIs in the presence of selection bias. Wang and Lee [19] also demonstrated that population structure may cause serious bias on estimated GEI effects in case-only studies. Recently, Dudbridge and Fletcher [20] showed that confounding due to population structure may cause dependence between gene and environment, and spurious GEIs can arise under this dependence. Although these studies provide useful information on theoretical impacts of population structure on GEI statistics, its influence in actual GxE GWASs has not been investigated comprehensively.

In this paper, we first show analytically that for the same reason that population structure causes spurious associations of genetic variants, it also causes spurious GEI associations based on the polygenic model. We show that disregarding sample structure can easily inflate test statistics for GEIs, leading to false positives. We then simulate a GxE GWAS using the 1000 Genomes Project dataset.[21] This simulation demonstrates the impact of population structure on GEI statistics more accurately than previous simulations because it is based on actual genotype data that resemble traditional GWAS datasets whereas previous simulations are not. We show that test statistics for GEIs as well as those for genetic variants are inflated due to population structure.

In addition to the simulation, we utilize two GxE GWAS datasets to show that population structure may cause serious effects on GEIs. One dataset is an expression quantitative trait loci (eQTL) study of the human aortic endothelia cell collected by Romanoski et al.[22] and Erbilgin et al.[23] Gene expression was collected with and without a certain treatment, which corresponds to an environmental exposure. The other dataset is a GWAS dataset of inbred mouse strains termed Hybrid Mouse Diversity Panel (HMDP) that consists of 100 classical inbred and recombinant inbred strains.[24] We analyze their lipid phenotypes, and the environment exposure is a thioglycollate injection to recruit macrophages. Both datasets are ideal for evaluating effect of population structure on GEI statistics for following reasons. First, it is known that population structure exists in both datasets; individuals in the human eQTL dataset are from multiple ethnicities, and mouse strains in the HMDP dataset have very diverse genetic backgrounds. Second, both datasets have many quantitative phenotypes to test effect of GEIs; the human eQTL dataset has gene expression measured at more than 18,000 probes, and the HMDP dataset has more than 20 different quantitative phenotypes. This variety of phenotypes allows us to comprehensively determine the impact of population structure on GEI statistics.

We also propose a statistical approach based on a linear mixed model to correct for population structure on GEI statistics. We show that the traditional mixed model approach [15] that incorporates genetic relatedness between individuals only corrects for population structure on effects of genetic variants and does not correctly control inflation of test statistics for GEIs. To solve this problem, we consider two types of pairwise similarities between individuals. One is the traditional genetic similarity that causes a pair of individuals who are genetically similar to have correlated phenotypes, and this causes inflation of test statistics on genetic effects. The other type of similarity is that individuals who are related and have the same environment or exposure status have similar phenotypes, which causes spurious GEIs. We extend the linear mixed model to take into account both types of similarities and show that our approach effectively removes inflation of test statistics for both GEIs and genetic variants in our simulation and the two GxE GWAS datasets.

## Results

### Spurious GEIs due to population structure using 1000 Genomes simulation

We generate a simulated GxE GWAS using two populations (GBR and TSI) of 1000 Genomes Project dataset.[21] Each population has 1,000 individuals whose genotypes are generated using only common variants found in a standard SNP chip. In this simulation, we consider a dichotomous environmental exposure and two scenarios; (1) each population has the same number of exposed and unexposed individuals and (2) one population has more exposed individuals than the other population. We generate the genetic kinship matrix (*K*) from genotype data and the GxE kinship matrix (*K*^*D*^) from *K* and the environmental exposure. Phenotypes are generated such that the genetic kinship (*K*) explains 40% of phenotypic variance while the GxE kinship (*K*^*D*^) explains 20% (See Materials and Methods). There is no causal variant in the simulation, meaning that the genomic control inflation factor (*λ*_*GC*_) should be close to one for both SNP and GEI statistics. We generate 100 replicates of simulation and measure inflation factors on SNP and GEI statistics of three different approaches. The first approach is one with no population structure correction on both SNP and GEI statistics (“OLS”), and another approach is a linear mixed model approach that incorporates the genetic kinship and accounts for population structure only on SNP statistics (“one RE”). The last approach is our proposed mixed model approach that uses both genetic and GxE kinship to correct for population structure on both SNP and GEI statistics (“two RE”).

**Fig 1:**
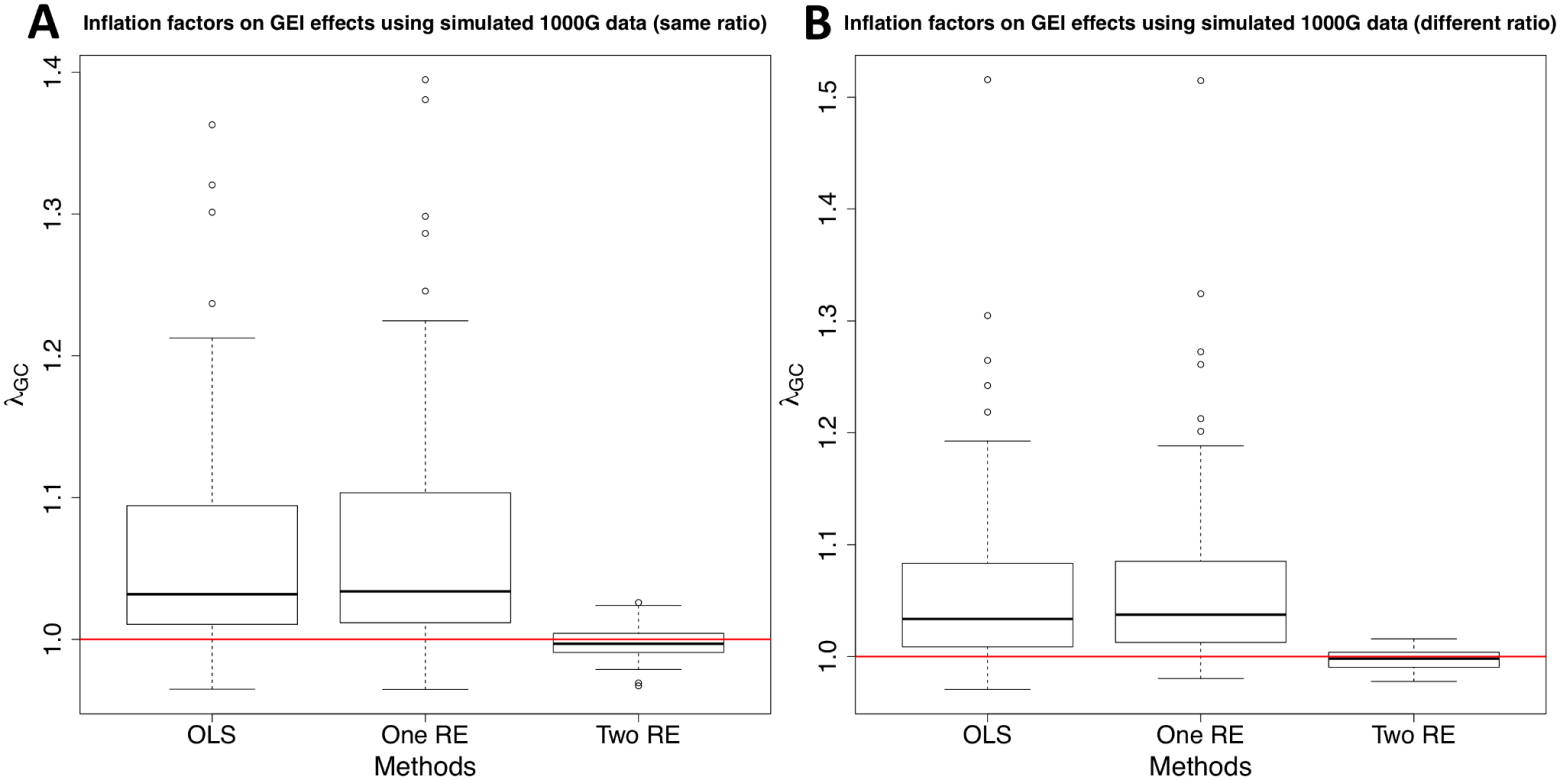
A distribution of inflation factors of GEI statistics on simulated 1000 Genomes data. We simulate genotype data using two populations (GBR and TSI), and genetic kinship (*K*) and GxE kinship (*K*^*D*^) explain 40% and 20% of phenotypic variance, respectively. We generate 100 replicates of simulation, and measure inflation factors of three methods for each replicate; OLS, One RE, and Two RE. Y-axis is the inflation factor, and horizontal red line is drawn at *λ*_*GC*_ = 1. We assume a dichotomous environmental status where the two populations have the same number of exposed and unexposed samples (**A**) and where one population has more exposed samples than the other population (**B**).

Fig 1 shows that population structure may cause spurious GEI associations because inflation factors on GEI statistics are on average greater than one. When the number of exposed and unexposed individuals is the same for both populations, the median *λ*_*GC*_ of the OLS approach is 1.032 and as high as *λ*_*GC*_ = 1.363. The results are similar when the ratio of exposed and unexposed individuals is different between the two populations. Population structure in the presence of GEIs may also cause inflation of SNP statistics, and S1 Fig shows that test statistics for SNPs are inflated. Also, *λ*_*GC*_ on SNP statistics tend to be higher than that on GEI statistics; the median inflation factor on SNP statistics is about 1.12. One of the reasons is that the genetic kinship (*K*) captures more phenotypic variance than the GxE kinship (*K*^*D*^) does in this simulation. The result demonstrates that both SNP and GEI effects are susceptible to false associations due to population structure.

The result of the simulation also indicates that we need to incorporate both genetic and GxE kinship matrices into the linear mixed model to correct for population structure on SNP and GEI statistics. While the one RE approach that uses only genetic kinship reduces inflation of test statistics on SNPs (S1 Fig), it has almost the same or slightly worse inflation factors on GxE statistics than OLS (Fig 1). With our approach, *λ*_*GC*_ becomes very close to one; the median *λ*_*GC*_ values on GEI statistics are 0.9969 and 0.9982 when the ratio of exposed and unexposed individuals between the two populations is the same and different, respectively. The maximum *λ*_*GC*_ values are also 1.026 and 1.0158, respectively. Interestingly, inflation factors on SNP statistics after applying our approach are even better than those after applying one RE; the median *λ*_*GC*_ with two RE is 0.9926 while that with one RE is about 1.02 when the ratio of exposed and unexposed individuals is the same. Hence, this shows that incorporating both kinship matrices also reduces inflation of test statistics on SNPs.

### Human eQTL GxE GWAS results

To assess the influence of population structure on a real GxE GWAS, we first analyze the eQTL study of human aortic endothelial cell (HAEC).[22, 23] Erbilgin et al. measured gene expression levels of 147 individuals with and without the oxidized phospholipid species, oxidized 1-palmitoyl-2-arachidonoyl-snglycero-3-phosphatidylcholine (Ox-PAPC) treatment. In order to have independent samples, to perform a GxE GWAS, we randomly selected 74 samples where we only used the treated samples and 73 samples where we only used the untreated samples. Due to the normality assumption of the linear regression model, we filter out probes of gene expression that do not follow the normal distribution and choose 8,720 probes for our analysis (See Materials and Methods). We also perform the same quality control as in the original paper for the genotype data, and about 575,000 SNPs are included in our analysis. We compute *λ*_*GC*_ for each probe on SNP and GEI statistics of the three methods as in the previous simulation.

**Fig 2:**
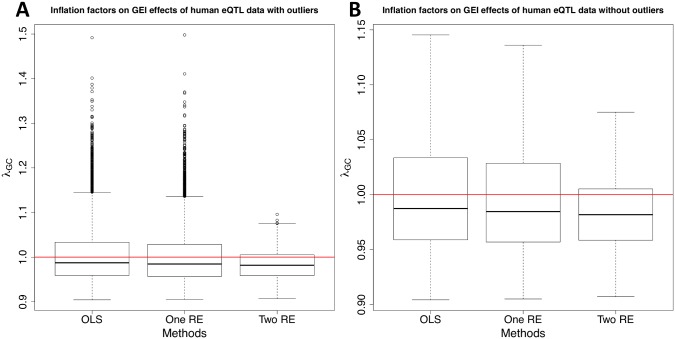
A distribution of inflation factors of GEI statistics on human eQTL GxE GWAS data. After filtering out probes whose expression values do not follow the normal distribution, 8,666 probes are tested for associations with about 500,000 SNPs. Gene expression of each individual was measured with and without the Ox-PAPC treatment, which corresponds to the environmental exposure. About a half of individuals were chosen to represent samples exposed to the environment, and the rest of individuals represent samples unexposed to the environment. We compute the inflation factor for each probe and for each of the three methods. Boxplots are drawn with outliers (**A**) and without outliers (**B**).

Fig 2 shows the distribution of inflation factors on GEI statistics with (Fig 2A) and without (Fig 2B) outliers. The results show that population structure indeed causes inflation of test statistics for GEIs, and our method can effectively correct for population structure in a real GxE GWAS. Although all three approaches have very similar median inflation factors for GEI statistics (0.98), OLS and one RE approaches have many more probes whose *λ*_*GC*_ values are greater than one than our approach. There are 2,687 (31% of total probes) and 2,509 (29%) probes with *λ*_*GC*_ > 1.02 according to OLS and one RE approaches, respectively, and the maximum *λ*_*GC*_ values are 1.492 and 1.498, respectively. After applying our approach, there are only 950 probes (11% of total probes) with *λ*_*GC*_ > 1.02 and the maximum is 1.096. Fig 2B shows that even after removing outliers from the plot, our method has a narrower range of inflation factors than OLS and one RE approaches do. S2 Fig shows that our method also reduces inflation of test statistics on SNPs. Most of probes whose *λ*_*GC*_ values on SNP statistics are around or greater than 1.4 in the OLS approach have *λ*_*GC*_ < 1.4 after applying our method although the median *λ*_*GC*_ of our method is greater than one (1.0365).

**Fig 3:**
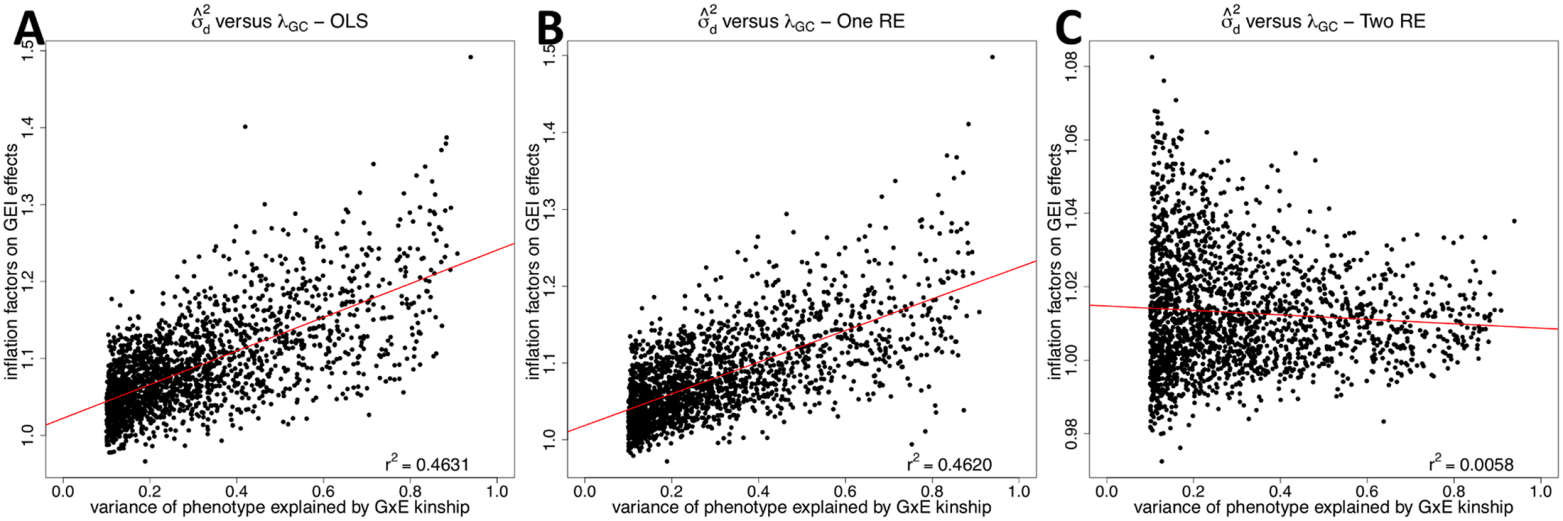
A correlation between the variance of phenotype explained by the GxE kinship matrix (${\hat{\sigma }}_{d}^{2}$) and the inflation factor on GEI statistics (*λ*_*GC*_) for human eQTL GxE GWAS data. The correlation is plotted for the three methods; OLS (**A**), One RE (**B**), and Two RE (**C**). Each dot is each probe, and x-axis is ${\hat{\sigma }}_{d}^{2}$ and y-axis is *λ*_*GC*_. We estimate ${\hat{\sigma }}_{d}^{2}$ using the GCTA software, and only probes with ${\hat{\sigma }}_{d}^{2}>10\%$ are shown in the plots. The red line is a regression line between *λ*_*GC*_ and ${\hat{\sigma }}_{d}^{2}$, and Pearson correlation coefficient is indicated on the top right of the plot.

We then compare the correlation between *λ*_*GC*_ and the variance of phenotype explained by the GxE kinship, denoted as ${\hat{\sigma }}_{d}^{2}$. We estimate variance components ($\sigma_{g}^{2},\sigma_{d}^{2},\sigma_{e}^{2}$ in Eq (8)) using GCTA software [25], and we obtain the ratio of each variance component to the total phenotypic variance. We focus on only probes whose ${\hat{\sigma }}_{d}^{2}>10\%$ because they are the probes in which the GxE kinship explains a certain amount of phenotypic variance. We find that about 24% (2,065) of probes have ${\hat{\sigma }}_{d}^{2}>10\%$. Fig 3A shows that inflation factors of OLS on GEI statistics tend to increase as the variance of phenotype explained by the GxE kinship increases; *r*^2^ between *λ*_*GC*_ and ${\hat{\sigma }}_{d}^{2}$ is 0.4631. This is expected because when $\sigma_{d}^{2}$ is higher, GEI effects become more susceptible to false positives due to population structure. This is similar to higher *λ*_*GC*_ on SNP effects for phenotypes with higher $\sigma_{g}^{2}$. [15] *r*^2^ between ${\hat{\sigma }}_{d}^{2}$ and *λ*_*GC*_ of the one RE approach (0.4620) is similar to that of the OLS approach (Fig 3B), meaning that it does not correct for population structure on GEI statistics. However, after applying our approach, *r*^2^ becomes 0.0058 (Fig 3C). This means that even when the GxE kinship explains high phenotypic variance and hence population structure can easily confound GEI associations, our method can successfully correct for population structure.

### HMDP GxE GWAS results

Next, we utilize the HMDP GxE GWAS dataset [24] that consists of many inbred mouse strains with very different genetic backgrounds. This diversity creates severe population structure, which was shown to easily cause spurious associations of SNP effects. [26] Hence, this dataset allows us to measure the impact of strong population structure on GEI statistics. We analyze 23 lipid phenotypes measured in more than 700 samples, and we test associations of about 74,000 SNPs after QC with these phenotypes. Macrophage recruitment was simulated in mice by injecting thioglycollate solution, which corresponds to environmental exposure in a GxE GWAS. The percentage of samples that received the injection varies between 30% to 42% for different phenotypes. We apply the three methods to each phenotype and measure the inflation factors on SNP and GEI statistics.

**Fig 4:**
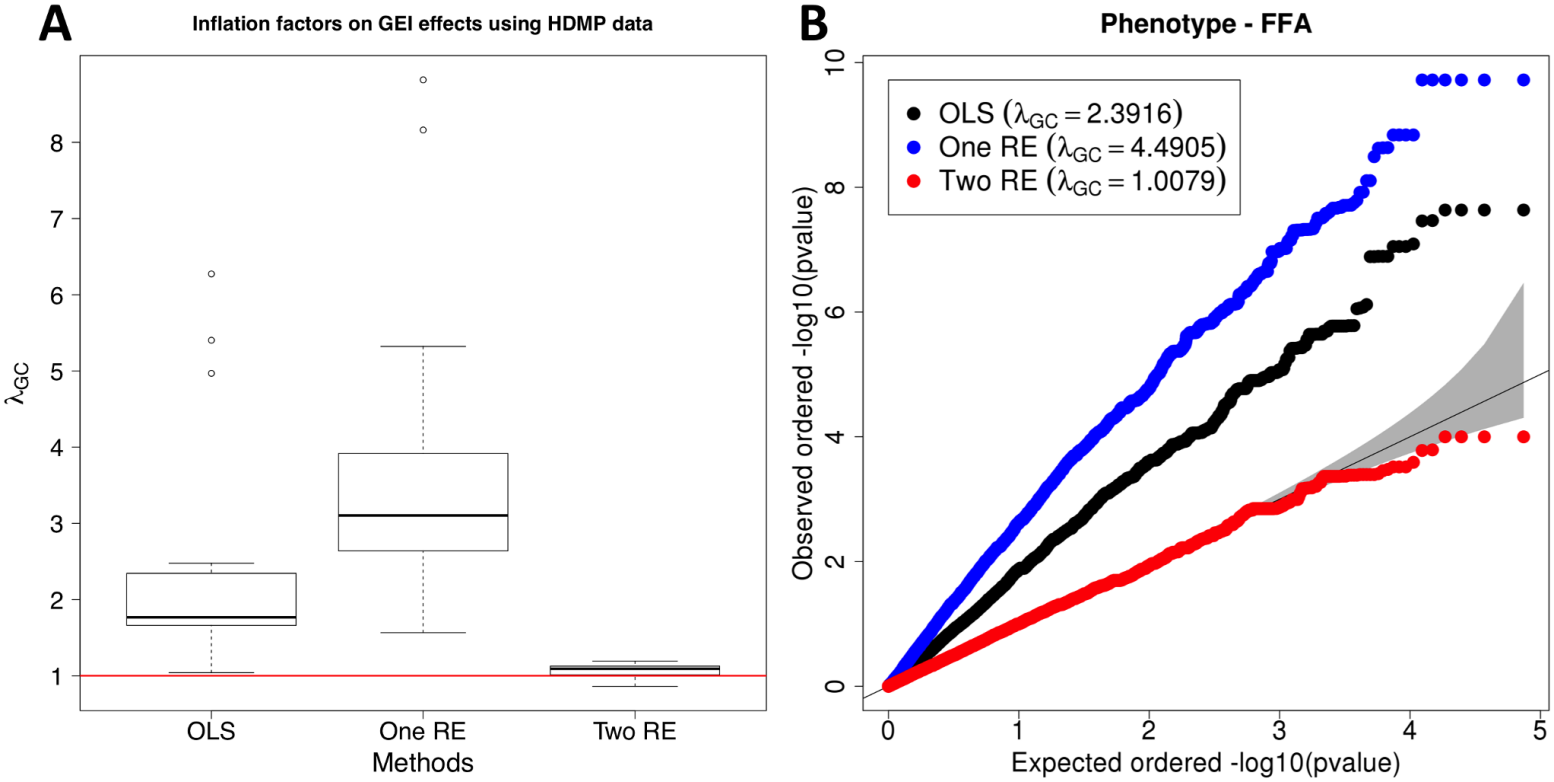
**A distribution of inflation factors of GEI statistics on HMDP GxE GWAS data (A).** HMDP consists of classical inbred strings and recombinant inbred strains. There are 23 lipid phenotypes, and about 74,000 SNPs are tested for associations. The environment is thioglycollate injection to recruit macrophages. We compute the inflation factor for each phenotype and for the three methods. (**B**) is a QQ plot of one of the phenotypes (free fatty acids, ffa), and it shows the distributions of p-values of GEI statistics for the three methods. Their inflation factors are indicated on the QQ plot.

Fig 4A shows that population structure causes serious inflation of test statistics for GEIs; the median inflation factor of the OLS approach is 1.77. Inflation factors of the HMDP dataset are generally much greater than those of the human eQTL dataset, and this is expected because the HMDP dataset has much stronger population structure effect than the human eQTL dataset does. The results also show that *λ*_*GC*_ value becomes close to one and more stable after applying our approach. The median *λ*_*GC*_ of two RE is 1.092, and especially the maximum *λ*_*GC*_ is 1.19, which is much smaller than 6.27 of the OLS approach. Interestingly, the one RE approach has a worse distribution of inflation factors than the OLS approach as both median and maximum *λ*_*GC*_ values of one RE are much greater than those of OLS. This result is to a certain degree consistent with results of the previous 1000 Genome simulation; one RE tends to have higher *λ*_*GC*_ than OLS in the 1000 Genomes simulation. The one RE model performs similarly to the OLS model which demonstrates that traditional mixed model methods do not correct for GxE interactions. In fact, the one RE model performed slightly worse than the OLS model which is likely because it is attempting to fix a statistical model which doesn’t fit the data. Fig 4B is a QQ plot of one of the phenotypes, free fatty acids (ffa), and it shows that test statistics for GEIs from our method follow the expected distribution while those from the two other methods clearly have inflation of test statistics. S3 Fig shows *λ*_*GC*_ on SNP statistics, and the results are similar to those of the human eQTL dataset; both one RE and two RE approaches successfully removes inflation of test statistics on SNPs.

**Fig 5:**
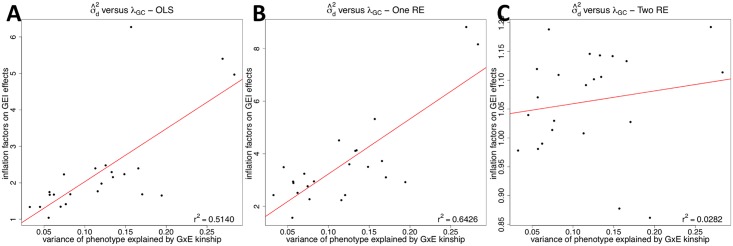
A correlation between the variance of phenotype explained by the GxE kinship matrix (${\hat{\sigma }}_{d}^{2}$) and the inflation factor on GEI statistics (*λ*_*GC*_) for HMDP GxE GWAS data. The correlation is plotted for the three methods; OLS (A), One RE (B), and Two RE (C). Each dot is each phenotype. The red line is a regression line between *λ*_*GC*_ and ${\hat{\sigma }}_{d}^{2}$, and Pearson correlation coefficient is indicated on the bottom right of the plot.

Table 1 lists the variance of phenotype explained by the genetic kinship matrix (${\hat{\sigma }}_{g}^{2}$), one by the GxE kinship matrix (${\hat{\sigma }}_{d}^{2}$) and inflation factors on GEI statistics for each phenotype. The genetic kinship matrix accounts for more phenotypic variance than the GxE kinship matrix for all phenotypes; the average ${\hat{\sigma }}_{g}^{2}$ is 50% while the average ${\hat{\sigma }}_{d}^{2}$ is 12%. However, for certain phenotypes, the GxE kinship explains more than 20% of phenotypic variance, and inflation factors on GEI statistics are greater for these phenotypes than for phenotypes with lower ${\hat{\sigma }}_{d}^{2}$. Fig 5 shows the correlation between *λ*_*GC*_ and ${\hat{\sigma }}_{d}^{2}$, and the OLS (Fig 5A) and one RE (Fig 5B) approaches have high correlations, which is similar to the results of the human eQTL dataset. However, our approach significantly reduces the correlation between ${\hat{\sigma }}_{d}^{2}$ and *λ*_*GC*_ (Fig 5C) meaning that our approach effectively removes effect of population structure.

**Table 1:** Variance of phenotype explained by the genetic kinship matrix (${\hat{\sigma }}_{g}^{2}$), variance of phenotype explained by the GxE kinship matrix (${\hat{\sigma }}_{d}^{2}$) and inflation factors for the three methods on GEI statistics for each phenotype of HMDP GxE GWAS data. Full name of each phenotype is discussed in Material and Methods section. GCTA software is utilized to estimate the phenotypic variance and its standard error for each phenotype.

Phenotype	Variance explained by *K*		Variance explained by *K*^*D*^		Inflation factor on GEI statistics		
	${\hat{\sigma }}_{g}^{2}$	SE	${\hat{\sigma }}_{d}^{2}$	SE	OLS	One RE	Two RE
bw	61.10%	4.55%	8.23%	2.29%	1.6859	2.9499	1.1093
fat_mass	60.43%	4.93%	13.30%	3.13%	2.2928	4.1107	1.1432
ffa	41.12%	5.81%	11.30%	3.66%	2.3959	4.5087	1.0079
ffp	45.00%	6.76%	28.34%	5.58%	4.9688	8.1618	1.1137
ffp_percentage	41.03%	6.69%	26.90%	5.43%	5.4027	8.8199	1.1924
free_fluid	36.00%	5.01%	7.68%	2.62%	1.4134	2.2710	1.0297
gfp	58.07%	5.35%	16.57%	3.75%	2.3959	3.7234	1.1332
gfp_percentage	58.91%	5.18%	14.86%	3.47%	2.2350	3.5021	1.1419
glucose_lc	35.82%	5.85%	17.05%	4.17%	1.6833	3.1030	1.0276
glucose	34.48%	6.16%	19.43%	4.63%	1.6512	2.9215	0.8614
hdl	60.82%	4.39%	4.51%	1.67%	1.3387	3.4900	1.0396
ldl_and_vldl	40.63%	5.02%	6.21%	2.35%	1.6759	2.5118	0.9900
lean_mass	66.21%	4.16%	7.04%	1.99%	1.3440	3.2399	1.1883
mfp	48.07%	5.37%	12.05%	3.21%	1.9778	2.4283	1.1458
mfp_percentage	44.87%	5.39%	11.59%	3.19%	1.7668	2.2345	1.0916
nmr_bf_percentage	61.60%	4.88%	13.45%	3.13%	2.1546	4.1319	1.1058
nmr_total_mass	55.82%	5.10%	12.56%	3.08%	2.4778	3.6011	1.1017
rfp	53.52%	4.79%	5.67%	2.03%	1.7447	2.9424	1.0703
rfp_percentage	48.75%	4.93%	5.70%	2.12%	1.6711	2.8876	0.9809
spleen_wt	52.11%	4.93%	5.58%	2.14%	1.0402	1.5656	1.1195
tc	65.33%	4.05%	3.26%	1.34%	1.3364	2.4279	0.9781
tg	34.53%	5.72%	15.68%	4.00%	6.2736	5.3221	0.8774
uc	57.35%	4.99%	7.45%	2.59%	2.2306	2.7644	1.0137

### 1-Random Effect Methods are often outperformed by OLS under significant popualtion structure

In Fig 4, we observe an anomalous result in HMDP GWAS data; the OLS results significantly *outperform* the 1RE method. This is somewhat surprising which computes a single variance component to account for the genetic heritability of the trait.

To determine why this is the case, we compare the amount of variance explained by *K*_*G*_ in the HMDP data with and without the use of a GxE study. To explore this, we simulated a population using the population structure derived from the HMDP data, as well as a population with a population with structure derived from our 1000G data. In this simulated data, we implanted several GxE associations and used GCTA to estimate the variance components of the model. These numbers are demonstrated in S1 Table. In this simulated data, we implanted several GxE associations and used GCTA to estimate the variance components of the model. We show that in the 1RE model, the variance components are substantially overestimates. We suspect that this is the cause of the higher observed inflation in the p-values compared to the OLS method.

### Simulation framework of population structure on GEIs

To simulate a gene-by-environment (GxE) GWAS with population structure, we utilize HAPGEN2 software [27] to generate genotype data of two populations in 1000 Genomes Project [21]; GBR (British in England and Scotland) and TSI (Toscani in Italy). We use only common variants in chromosomes 11, 12, 13, and 14 whose minor allele frequency is greater than 5% in both populations. We also use variants present in the Illumina OmniExpress 730K genotyping chip to simulate a typical GWAS. The number of SNPs after this filtering is 99,612, and we generate 1,000 individuals for each population for a total of 2,000 individuals.

To generate phenotype values, we sample them from the following multivariate normal distribution.

$$y∼N\left(0,\sigma_{g}^{2}K+\sigma_{d}^{2}K^{D}+\sigma_{e}^{2}I\right)$$

We create a genetic kinship matrix (or genetic relationship matrix) **K** using the GCTA software from the simulated genotype data. To create a GxE kinship matrix **K**^**D**^, we first need to assign an environmental exposure to each individual. We assume a dichotomous variable where a half of individuals (1,000) are exposed and the rest (1,000) are unexposed. When assigning an environmental exposure to each sample, we consider two possible cases. One is that each population has the same number of exposed and unexposed individuals. In other words, each population has exactly 500 exposed and 500 unexposed individuals. The other case is that one population has more exposed than unexposed individuals. For example, the ratio between exposed and unexposed in one population is 0.6 while it is 0.4 in other population. This is possible in actual GxE GWASs when individuals in one population are more easily exposed to the environment than those in other population. We vary this ratio from 0.54 to 0.6 in one population. We consider both cases in our simulation to determine how they influence results. Once we decide on the number of exposed and unexposed for each population, we randomly assign the environmental exposure to each individual and create the GxE kinship matrix. The phenotype values are generated such that the genetic kinship matrix and GxE kinship matrix explain 40% and 20% of phenotypic variance, respectively. In other words, $\sigma_{g}^{2}$ is 0.4, $\sigma_{d}^{2}$ is 0.2 and $\sigma_{e}^{2}$ is 0.4. We generate 100 replicates of this simulation.

### Simulations of low $\sigma_{d}^{2}$

We also simulated our method at values of $\sigma_{d}^{2}$ (*i.e.* low GxE kinship) between 0 and 0.2, while holding the ratio between $\sigma_{g}^{2}$ and $\sigma_{e}^{2}$ constant; results are shown in S4 Fig. There appears to be an approximately linear relationship between the amount of inflation (*λ*_*GC*_) and the size of $\sigma_{d}^{2}$. As less of the total variance is explained by the GxE kinship, the 2RE method begins to deflate p-values slightly, while both the 1RE and OLS methods improve. However, the over-correction of 2RE methods is small, and near $\sigma_{d}^{2}=0$, 1RE and 2RE methods perform similarly.

### Bias and variance of $\hat{\sigma_{d}^{2}}$

Because the sample sizes in the HMDP and HAEC datasets are rather low for GWAS, we wanted to demonstrate that our method can accurately detect the amount of GxE kinship.

In order to do this, we performed simulations of populations with the same genetic kinship matrix as was computed from the HMDP dataset, and a phenotype distributed with an implanted GxE kinship component. The results, which demonstrate accurate estimation of this GxE component are in S6 Fig.

### Principal components x environment studies

A alternate approach for correcting for population structure in gene-by-environmental interaction studies is to include principal components of the genetic relatedness matrix as covariates similar to the approach of EIGENSTRAT. We can extend such approaches to the scenario of gene-by-environment interactions by adding additional covariates of the form of the principal component times the environmental covariate (PCs × environment). We find that using PCs × environment does reduce inflation, but at a lesser extent than including a specifically GxE-based kinship matrix. This is consistent with comparisons of mixed models and principal components in traditional association studies [15]. We demonstrate these results in S7 Fig.

### Effect of quantile normalization of phenotypes

Many of the phenotypes explored in the HMDP and HAEC datasets are close to normally-distributed but are not completely normal. We examine if this is a source of inflation by quantile normalizing the data. In S5 Fig, we observe that quantile normalization of the phenotypes does improve the performance of 1RE and OLS methods, but only slightly.

## Discussion

We demonstrated that population structure may cause spurious associations of gene-by-environment interactions. Using the same argument that population structure can inflate test statistics for genetic variants in the polygenic model, we were able to derive analytically that the same phenomenon may occur for GEI statistics. We then used the 1000 Genomes simulation and two GxE GWAS datasets to observe the impact of population structure on GEI statistics. When the severe population structure exists as in the mouse GxE GWAS dataset, we observed very high inflation factors for GEI statistics. When the influence of population structure is relatively moderate as in the 1000 Genome simulation and the human eQTL GxE GWAS dataset, we found that test statistics for GEIs are nonetheless inflated, which may cause spurious associations. While Wang et al.[17] showed through simulations that population structure may cause small biases to estimated GEI effects when there exists small correlation among environments and genetic variants, their results are not based on actual GxE GWAS datasets. Hence, our results that make use of current GxE GWASs may more accurately represent the impact of population structure on GEI statistics, and our results indicate that even moderate population structure may cause unacceptable inflation of test statistics for GEIs.

To correct for population structure on GEI statistics, we proposed a linear mixed model approach that includes two random effects to take into account two types of similarities between individuals. One is the genetic similarity, and the other is the similarity caused by both genetic and environment. By incorporating two kinship matrices corresponding to the two similarities into linear mixed models, we were able to correct for population structure on GEI statistics successfully. We showed that accounting for only the genetic similarity controls the inflation of test statistics for SNPs, but not for GEIs. This is important because GWASs typically include only the genetic kinship matrix to correct for population structure.[15] Sul and Eskin [16] proposed the idea of including two random effects in linear mixed models to account for two types of population structure; one caused by SNPs under selection and the other by rest of SNPs. As demonstrated in their and this papers, this approach is effective in removing inflation caused by two different types of population structure or confounding.

Recently, Zheng et al.[11] studied the roles of GEIs on type 2 diabetes (T2D) related traits and observed inflation of test statistics on GEIs. They collected information regarding to several dietary and lifestyle factors that may influence the T2D-related traits. These factors were considered as environmental exposure in their GxE analysis, and they measured the variance of T2D-related phenotype explained by GEIs for the different environmental factors. They also performed a GxE GWAS and observed that test statistics for GEIs were inflated for environmental factors that explained a significant amount of the phenotypic variance while they observed no inflation for factors that did not contribute to the phenotypic variance. One of the possible reasons for this inflation is population structure because they did not correct for population structure on GEI statistics. Their result is also consistent with our finding that the inflation factor is higher for a phenotype with higher ${\hat{\sigma }}_{d}^{2}$, the phenotypic variance explained by the GxE kinship. Hence, as more GxE GWASs are conducted to discover GEIs associated with traits, correcting for population structure will become important to reduce inflation of test statistics and to remove possible false positive associations.

The linear mixed model in our two RE approach is based on the GCTA GEI model [25], and we use GCTA software to estimate variance components. While previous GxE studies utilized GCTA software to estimate phenotypic variance explained by GEIs, to the best of our knowledge, they did not attempt to measure effect of population on GEIs and to correct for population structure. Our approach is the first method to use the linear mixed model with two kinship matrices to correct for population structure on both SNP and GEI effects.

In this paper, we mainly focused on inflation of test statistics for measuring GEI effects of the three different approaches. We showed that only two RE approach achieves correct false positive rate while the two other methods do not. When comparing performance of different statistical tests, it is also important to compare power of tests in addition to measuring false positive rates. However, power comparison can only be made when all tests achieve the correct false positive rates. In our simulation and real data, it is not possible to compare power among the three different methods because OLS and one RE have incorrect false positive rate.

We note that in our results, we are including the marker that we are testing in the kinship matrix. Recently, several studies have pointed out that including the tested marker in the kinship matrix effectively includes the marker twice in the statistical model and this is what causes the inflation factor to be one even when there are many genetic effects throughout the genome.[28] Our results are orthogonal to these approaches and the recommendation of those studies should apply to testing for GEI as well. Nevertheless, in our experiments, we decided to include the tested marker in the kinship because this more easily exposes inflation of test statistics since we expect to observe an inflation factor of one if there is no inflation.

## Materials and Methods

### Spurious genetic associations due to population structure in the polygenic model

Before we discuss how population structure may cause spurious GEI associations, we first review how it influences associations of genetic variants because the two concepts are closely related. We assume that genetic effects are additive and there are *M* variants. Then, the standard genotype-phenotype model is

$$y_{k}=\mu +\sum_{i=1}^{M}\beta_{i}X_{ik}+\varepsilon_{k}$$ (1)

where *y*_*k*_ is individual *k*’s phenotype value, *μ* is the mean of the phenotype, *X*_*ik*_ is the genotype of individual *k* at variant *i*, *β*_*i*_ is the effect of genetic variant *i*, and *ε*_*k*_ is the residual. The polygenic model assumes that there are many variants with small effects, which means that many *β*_*i*_’s are non-zero. The traditional association study considers each genetic variant individually and tests the effect of each genetic variant on the basis of the following model.

$$y_{k}=\mu +\beta_{r}X_{rk}+\eta_{\bar{r}k}$$ (2)

The goal of association studies is to identify the set of genetic variants with *β* ≠ 0 since these are variants that putatively affect the phenotype. Note that we use different notations for the residual terms (*ε*_*k*_ in Eq (1) and $\eta_{\bar{r}k}$ in Eq (2)) to emphasize the difference between the two residuals. The residual in Eq (2) in relation to Eq (1) is exactly

$$\eta_{\bar{r}k}=\sum_{M:i\neq r}\beta_{i}X_{ik}+\varepsilon_{k}$$ (3)

According to Eq (3), people who are related would have similar residual terms ($\eta_{\bar{r}k}$) because they share the same genotypes (*X*_*ik*_). This violates the assumption of the traditional linear regression model in Eq (2) that residuals are independent and hence causes bias in the estimation of *β*_*r*_. Therefore, sample structure in GWAS datasets such as population structure or cryptic relatedness may cause inflation of test statistics (*β*_*r*_) for genetic variants.[12, 29] For clarity, we refer to the statistics testing for the effect of a genetic variant as SNP statistics to distinguish from statistics testing for the presence of GEI which we refer to as GEI statistics.

One approach to account for the sample structure is through the use of a linear mixed model.[15, 26, 30, 31] This approach introduces a random effect into the linear model in Eq (2) to account for the global genetic relatedness resulting in the following model

$$y=\mu +\beta_{r}X_{r}+u+e$$ (4)

where **y** = [*y*_1_, *y*_2_, …, *y*_*n*_]^*T*^ and **X**_**r**_ = [*X*_*r*1_,*X*_*r*2_, … ,*X*_*rn*_]^*T*^ where *n* is the number of individuals. **u** is the random effect in the mixed model that captures effect of population structure, and $\text{var}(u)=\sigma_{g}^{2}K$ and $\text{var}(e)=\sigma_{e}^{2}I$ where **K** is an *n* × *n* kinship matrix and **I** is an identify matrix of size *n*. Then, the total variance of phenotype is given as $\text{var}(y)=\sigma_{g}^{2}K+\sigma_{e}^{2}I$. It has been shown that this linear mixed model approach that incorporates the pairwise genetic relatedness into the linear model effectively controls inflation of test statistics for genetic variants due to sample structure.[15, 26]

### Spurious gene-by-environment interactions due to population structure

We extend the standard model to consider an environmental factor *D*. For simplicity, we assume it is a dichotomous variable. The exposure of an individual *k* to the environmental factor is denoted as *D*_*k*_; *D*_*k*_ = 0 for the unexposed and *D*_*k*_ = 1 for the exposed. The model corresponding to Eq (1) is now

$$y_{k}=\mu +\sum_{i=1}^{M}\beta_{i}X_{ik}+\delta D_{k}+\sum_{j=1}^{M}\gamma_{j}D_{k}X_{jk}+\varepsilon_{k}$$ (5)

where *δ* represents the fixed effect of environmental factor *D* and each *γ*_*j*_ is the gene-by-environment interaction effect of variant *j* and environmental factor *D*. The goal of GxE association studies is to discover genetic variants or SNPs whose *γ*_*j*_ ≠ 0 because they have effects on the phenotype in the presence of the environmental factor. While it appears from Eq (5) that interaction effects only affect the phenotype when *D*_*k*_ = 1, this is not always the case. For example, when *β*_*i*_ = −*γ*_*j*_ and *D*_*k*_ = 1, a SNP does not influence the phenotype because SNP effects and interaction effects cancel each out. In this case, the SNP has effects on phenotype only for unexposed individuals (*D*_*k*_ = 0). Similar to the association study of genetic variants, we test the effect of each genetic variant and its GxE effect individually, which corresponds to fitting the following model.

$$y_{k}=\mu +\beta_{r}X_{rk}+\delta D_{k}+\gamma_{k}D_{k}X_{jk}+\tau_{\bar{r}k}$$ (6)

where $\tau_{\bar{r}k}$ is the residual. This residual is precisely

$$\tau_{\bar{r}k}=\sum_{M:i\neq r}\beta_{i}X_{ik}+\sum_{M:j\neq r}\gamma_{j}D_{k}X_{jk}+\varepsilon_{k}$$ (7)

These residuals ($\tau_{\bar{r}k}$) are not independent if individuals are related. For people who are genetically similar, they would have similar value for the first sum (∑_*M*: *i*≠*r*_ *β*_*i*_ *X*_*ik*_), and people who are genetically similar and are in the same environment, they would have similar value for the second sum (∑_*M*: *j*≠*r*_ *γ*_*j*_ *D*_*k*_ *X*_*jk*_). Hence, this equation shows that for the same reason population structure causes spurious genetic associations as shown in Eq (2), it may inflate test statistics of GEIs and cause false positive associations due to correlated residuals.

### Linear mixed model to correct for population structure on GEIs

We extend the linear mixed model approach to correct for population structure on GEI statistics by introducing an additional random effect that captures the similarity of individuals due to GEI effects. Given the kinship matrix (*K*), we define the matrix *K*^*D*^ where each entry $K_{ij}^{D}=K_{ij}$ if *D*_*i*_ = *D*_*j*_ and $K_{ij}^{D}=0$ otherwise.[25] This matrix *K*^*D*^ describes how individuals are related both genetically and environmentally because a pair of individuals who are genetically related and share the same environment exposure have a non-zero kinship coefficient. We name *K*^*D*^ “GxE kinship” and *K* “genetic kinship” to distinguish two kinship matrices. We propose the linear mixed model that incorporates both kinship matrices as following

$$y=\mu +\beta_{r}X_{r}+\delta D+\gamma_{r}D\cdot X_{r}+u+v+e$$ (8)

where **D** = [*D*_1_,*D*_2_, … ,*D*_*n*_]^*T*^ is a column vector of environmental exposures and **D**⋅**X**_**r**_ is the element-wise product. The random effect **v** accounts for the relatedness of individuals due to GEI effects and $\text{var}(v)=\sigma_{d}^{2}K^{D}$. The total variance of **y** is then given as $\text{var}(y)=\sigma_{g}^{2}K+\sigma_{d}^{2}K^{D}+\sigma_{e}^{2}I$. We call this approach “**two RE**” because it uses two random effects to correct for population structure on both SNP and GEI statistics.

We compare our approach to other approaches that do not consider effect of population structure on GEI statistics. One such approach is a simple linear regression without any random effect. We name this approach “**OLS**” from ordinary least squares, and it is defined as

$$y=\mu +\beta_{r}X_{r}+\delta D+\gamma_{r}D\cdot X_{r}+e$$ (9)

Note that this does not correct for population structure either on SNP statistics or on GEI statistics. Another approach is to correct for population structure only on SNP statistics by including one random effect that accounts for genetic relatedness. Its model is

$$y=\mu +\beta_{r}X_{r}+\delta D+\gamma_{r}D\cdot X_{r}+u+e$$ (10)

This approach would account for the similarity due to genetic effects (the first sum in Eq (7)), but would not correct for the similarity due to GEI effects (the second sum in Eq (7)). This is because it is likely that values for *β*_*i*_ and *γ*_*j*_ are different for each variant, and the random effect **u** would not capture GEI effects which is the second sum in Eq (7). We name this approach “**one RE**” because it uses only one random effect.

P-values of all three approaches can be estimated using a standard *F*-test. Let *Σ* = **I** for OLS, $\Sigma ={\hat{\sigma }}_{g}^{2}K+{\hat{\sigma }}_{e}^{2}I$ for one RE, and $\Sigma ={\hat{\sigma }}_{g}^{2}K+{\hat{\sigma }}_{d}^{2}K^{D}+{\hat{\sigma }}_{e}^{2}I$ for two RE where ${\hat{\sigma }}_{g}^{2},{\hat{\sigma }}_{d}^{2},{\hat{\sigma }}_{e}^{2}$ are estimated variance components. We utilize GCTA software [25] to estimate these variance components ($\sigma_{g}^{2},\sigma_{d}^{2},\sigma_{e}^{2}$), and we estimate them for each phenotype once and apply them for all SNPs. This is the same as the EMMAX approach [15], and this approach markedly reduces the computational time while maintaining the similar power to that of an approach that estimates variance components for each SNP.

Let *β* include effects of all covariates in the linear regression model that includes a SNP effect (*β*_*r*_) and a GEI effect (*γ*_*r*_) and let **X** include all covariates including a SNP (**X**_**r**_) and a GEI (**D**⋅**X**_**r**_). Then, the estimated *β* is

$$\hat{\beta }={\left(X^{\prime }\Sigma^{-1}X\right)}^{-1}X^{\prime }\Sigma^{-1}y$$ (11)

We perform a standard *F*-test for the null hypothesis *β*_*r*_ = 0 and *γ*_*r*_ = 0 to obtain p-values for SNP and GEI effects, respectively. We provide a software package that implements the two RE approach at http://genetics.cs.ucla.edu/pylmm/. Our approach can efficiently be applied to standard GWAS datasets that contain thousands of individuals and hundreds of thousands of SNPs, similar to the linear mixed models for GWAS [15].

### Exploring GEI of continuous environmental factors

It is very attractive to extend this model to the case of continuous covariates; however, it is not straightforward to create *K*^*D*^ from *K*. Under a binary enviornmental exposure, setting $K_{i,j}^{D}=K_{i,j}\times \delta_{D_{i},D_{j}}$ where *δ*_*D*_*i*_,*D*_*j*__ is 1 if the environmental exposures of indivdiuals *i* and *j* are the same and 0 otherwise is intuitive. For continuous covariates, we can extend this formalism to: $K_{i,j}^{D}=K_{i,j}\times f\left(D_{i},D_{j}\right)$ for some function *f*. A good general guideline for a choice of function is to use *f* = 1−*d*(*D*_*i*_,*D*_*j*_) where *d* is a metric with range [0, 1]. Two of the most natural choices that satisfy the above recommendation are: $f\left(i,j\right)=1-|\frac{D_{i}-D_{j}}{R}|$, where *R* is the range of the environmental exposures, or $f\left(i,j\right)=1-|\Phi (\frac{D_{i}-\mu_{D}}{\sigma_{D}})-\Phi (\frac{D_{j}-\mu_{D}}{\sigma_{D}})|$, where *μ*_*D*_ and *σ*_*D*_ are the mean and standard deviation of the environmental exposures, and **Φ** is the standard normal cumulative distribution function. However, the best choice of *f* will depend on the scale of the environmental exposure.

### Human eQTL GxE GWAS dataset

Erbilgin et al.[23] performed the expression quantitative trait loci (eQTL) study of human aortic endothelial cell (HAEC). They collected HAEC cultures from 147 unrelated heart transplant donors, and the oxidized phospholipid species, oxidized 1-palmitoyl-2-arachidonoyl-snglycero-3-phosphatidylcholine (Ox-PAPC) treatment was applied to the cells. It has been known that Ox-PAPC promotes vascular inflammation and regulates more than 1,000 transcripts in this cell type.[22, 32] Gene expression was collected both with and without the Ox-PAPC treatment. In order to have the two conditions have independent samples, we randomly chose a subset of 74 individuals where we only used the treated samples and a different 73 individuals where we only used the untreated samples, which represents two exposure statuses of environment.

The gene expression on 18,630 probes is collected using Affymetrix HT HG-U133A microarrays. The COMBAT software was utilized to correct for batch effects in the expression data [33]. Since the linear regression model assumes that expression values follow the normal distribution, we filtered out probes whose Shapiro-Wilk test p-values are less than 0.05. Additionally, we computed the number of outliers for each probe whose expression values are two standard deviations apart from the mean, and excluded probes containing five or more outliers (5% of total samples). These filters removed 9,910 probes, leaving 8,720 probes for the subsequent analysis. To verify that our results are not affected by the fact that the data still deviates from the normal distribution, we reanalyzed the data after performing quantile normalization both within each exposure group and over the entire dataset. In these additional experiments we observed equivalent results as shown in S5 Fig.

SNPs are genotyped using Affymetrix Genome-Wide Human SNP Array 6.0. We used the same QC filters as in the original paper [23]: MAF of 10%, HWE p-value of 10^−4^, and genotype completeness of 5%, and 575,042 SNPs in autosomes are tested for associations. Erbilgin et al. performed a principal component analysis to identify population structure among the 147 individuals with 11 HapMap3 populations and found that there are groups of individuals with different ethnicities.

### HMDP GxE GWAS dataset

Hybrid Mouse Diversity Panel (HDMP)[24] consists of 100 inbred strains including 29 classic inbred strains and three sets of recombinant inbred strains. The 23 lipid phenotypes (and their abbreviations) that were analyzed are: body weight (bw), fat mass by NMR (fat_mass), free fatty acids (ffa), Femoral fat pad (ffp), Femoral fat pad/total bw (ffp_percentage), water weight by NMR (free_fluid), gonadal fat pad (gfp), gonadal fat pad/total bw (gfp_percentage), glucose at time for sac (glucose), glucose by lipid core (glucose_lc), HDL (hdl), LDL and VLDL (ldl_and_vldl), lean mass by NMR (lean_mass), mesenteric fat pad (mfp), mesenteric fat pad/total bw (mfp_percentage), Body fat percentage determined by NMR (nmr_bf_percentage), total mass by NMR (nmr_total_mass), retroperitoneal fat pad weight (rfp), retroperitoneal fat pad weight/total bw (rfp_percentage), spleen weight (spleen_wt), total cholesterol (tc), triglycerides (tg), unesterified cholesterol (uc). The number of samples from the 100 inbred strains that are phenotyped varies between 735 and 894 among these phenotypes. These strains are genotyped at more than 130,000 SNPs, and we applied following QC; genotype completeness of 98% for both SNPs and individuals and minor allele frequency threshold of 10%. After the QC, we have about 74,000 SNPs for performing association studies. The environment that we are interested in is thioglycollate injection to recruit macrophages. Macrophages play an important role in inflammatory component of many common diseases.[34] The percentage of exposed samples is between 30% and 42% depending on phenotypes. Note that individuals from the same strain can be both exposed and unexposed.

## Supporting Information

S1 Fig A distribution of inflation factors on SNP statistics on simulated 1000 Genomes data. Note that the scale is different from Fig 1. In **A**, the same number of exposed an unexposed individuals were generated in each simuation; in **B**, fewer exposed individuals were simulated compared to unexposed individuals.
(TIF)

S2 Fig A distribution of inflation factors of SNP statistics on human eQTL GxE GWAS data. Note that the scale is different from Fig 2.
(TIF)

S3 Fig A distribution of inflation factors of SNP statistics on HMDP GxE GWAS data. Note that the scale is different from Fig 4A.
(TIF)

S4 FigA distribution of the median inflation factors for SNP statistics on simulated 1000G GxE GWAS.The GxE variance $\sigma_{d}^{2}$ is taken from 0 to 0.2 while the purely genetic variance $\sigma_{g}^{2}$ and the purely random variance $\sigma_{e}^{2}$ are held constant.(TIF)

S5 Fig We explore the effect of quantile normalization between groups and within groups compared to the OLS estimate by itself. In **A**, we apply no quantile normalization; in **B**, the whole sample is first quantile normalized; in **C**, each environmental group is quantile-normalized separately. There is an apparent increase in performance of OLS and 1RE methods (their *λ*_*GC*_ becomes closer to 1) using quantile normalization, however, the performance is not as good as 2RE methods. There is little appreciable difference between the population-wide quantile normalization and within-group quantile normalization.
(TIFF)

S6 FigWe demonstrate our method’s ability to accurately measure implanted $\sigma_{d}^{2}$ through simulation.The bias is low, and the variance of the estimate appears to be somewhat proportional to the size of $\sigma_{d}^{2}$.(TIF)

S7 Fig We compare the performance of our 2-RE method with methods using a single random effect, regressing out the specified number of top principal components of the GxE kinship matrix from the output data. (TIF)

S1 TableVariance of phenotype explained by the genetic kinship matrix (${\hat{\sigma }}_{g}^{2}$), when estimated in the absence of a GxE effect.The GxE heritability is highly significant for many phenotypes in Table 1, which this table references for the variances explained. GCTA software, using the environmental factor as a covariate, is utilized to estimate the phenotypic variance and its standard error for each phenotype.(PDF)
