# Supplementary figures and images for: Accounting for Population Structure in Gene-by-Environment Interactions in Genome-Wide Association Studies Using Mixed Models
Source: PLoS Genet. 2016 Mar 4;12(3):e1005849. doi: 10.1371/journal.pgen.1005849 (PMC4778803; doi:10.1371/journal.pgen.1005849)

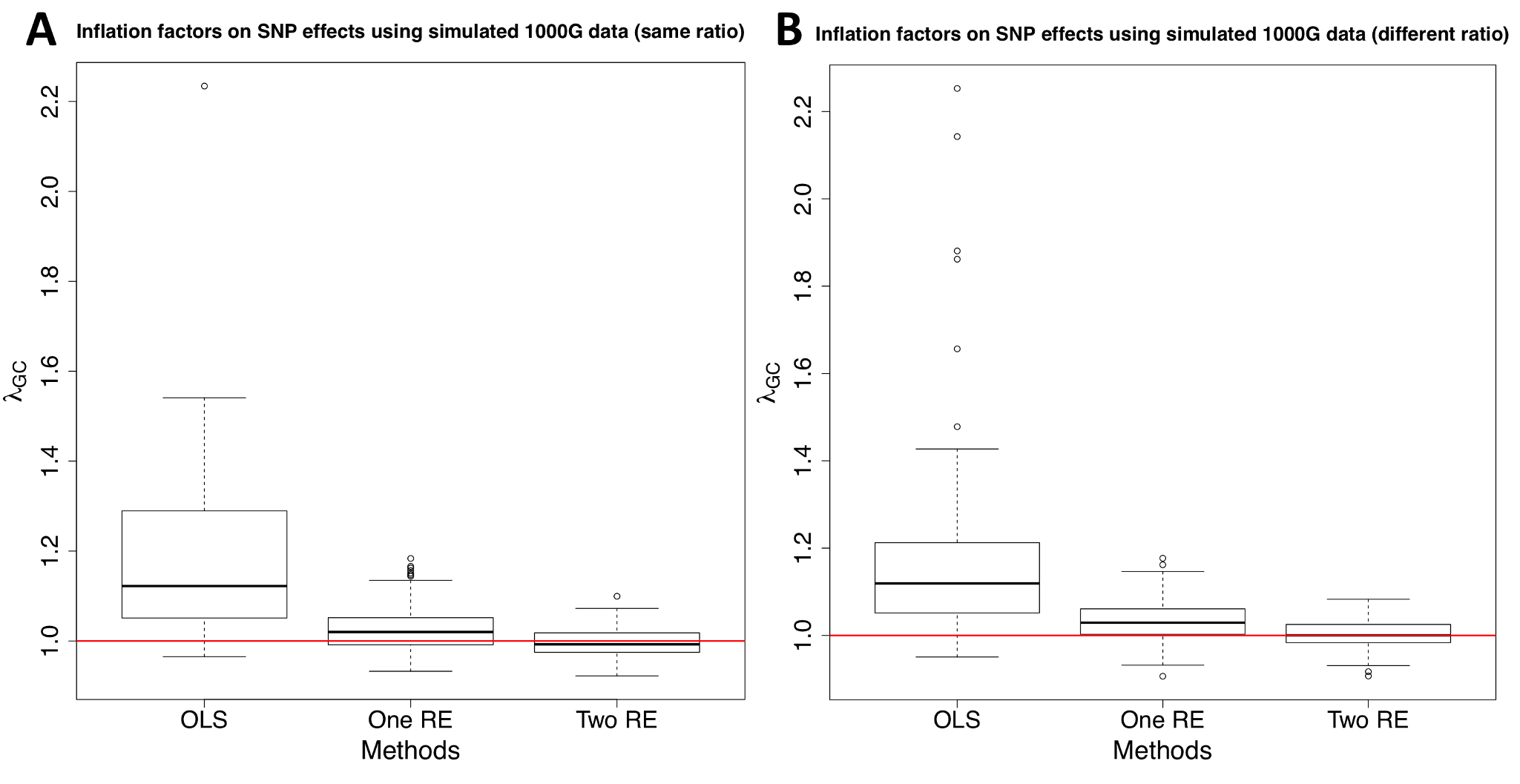

Supplement: S1 Fig — Note that the scale is different from Fig 1. In A, the same number of exposed an unexposed individuals were generated in each simuation; in B, fewer exposed individuals were simulated compared to unexposed individuals. (TIF) [file pgen.1005849.s001.tif]

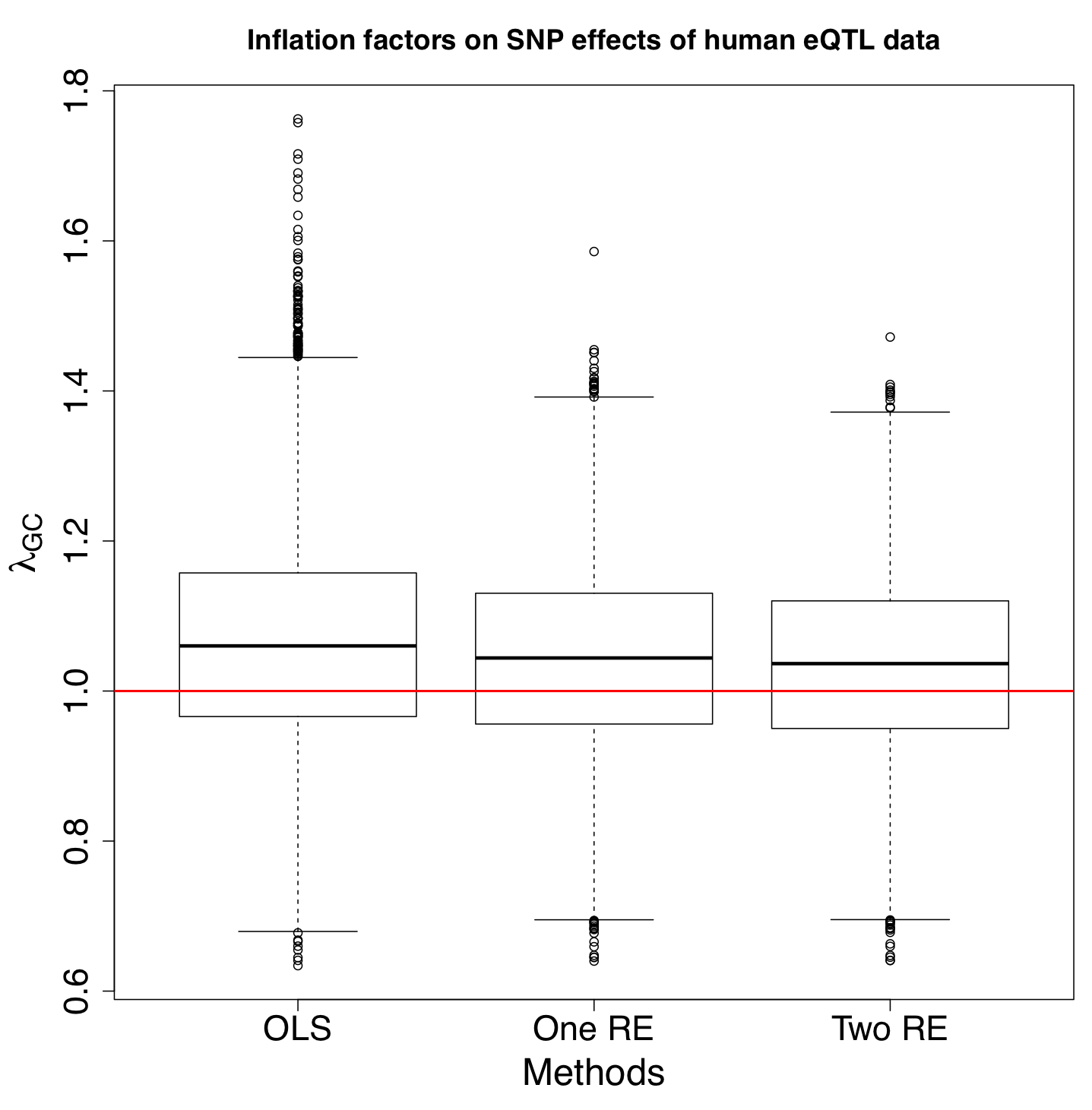

Supplement: S2 Fig — Note that the scale is different from Fig 2. (TIF) [file pgen.1005849.s002.tif]

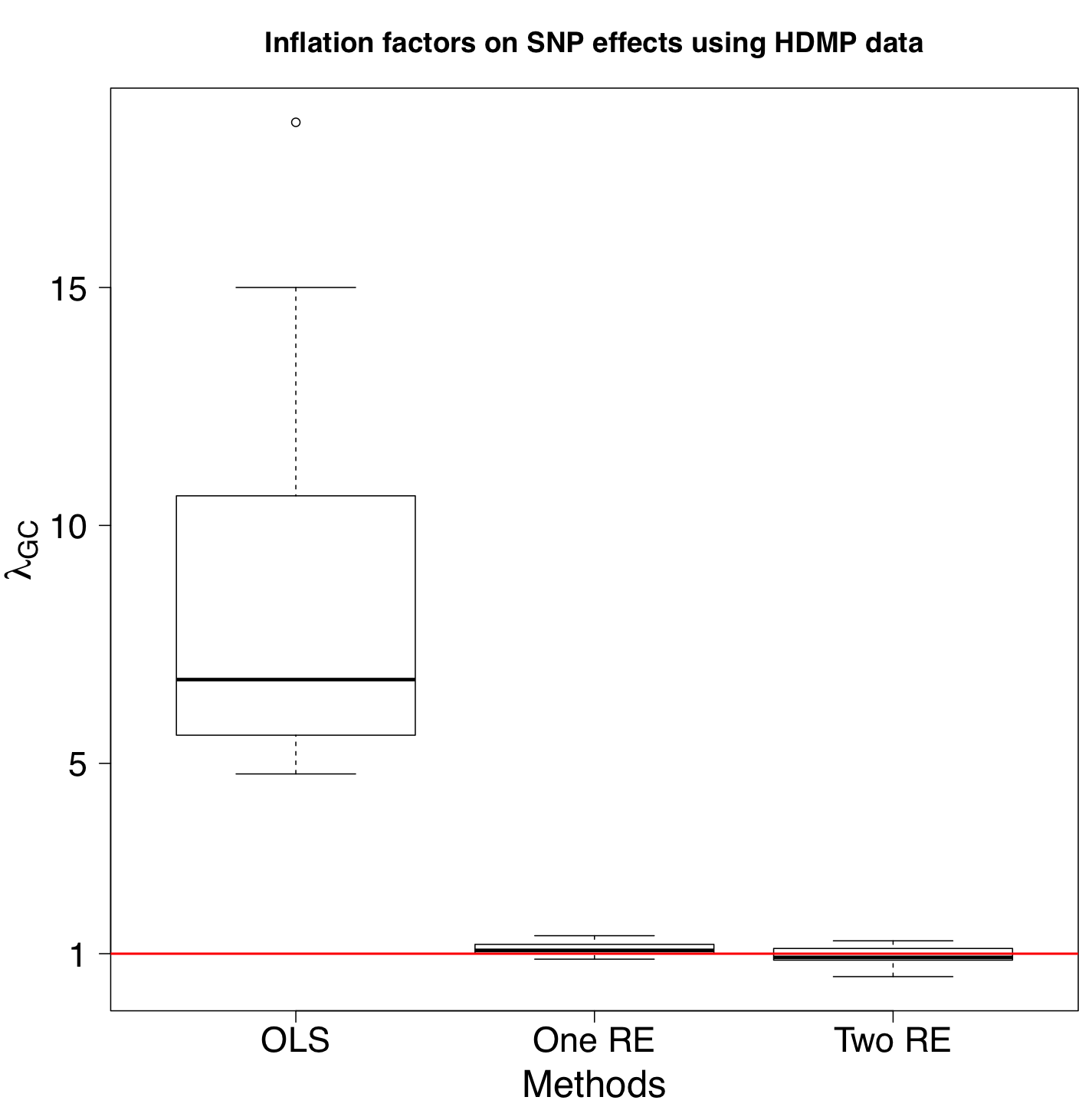

Supplement: S3 Fig — Note that the scale is different from Fig 4A. (TIF) [file pgen.1005849.s003.tif]

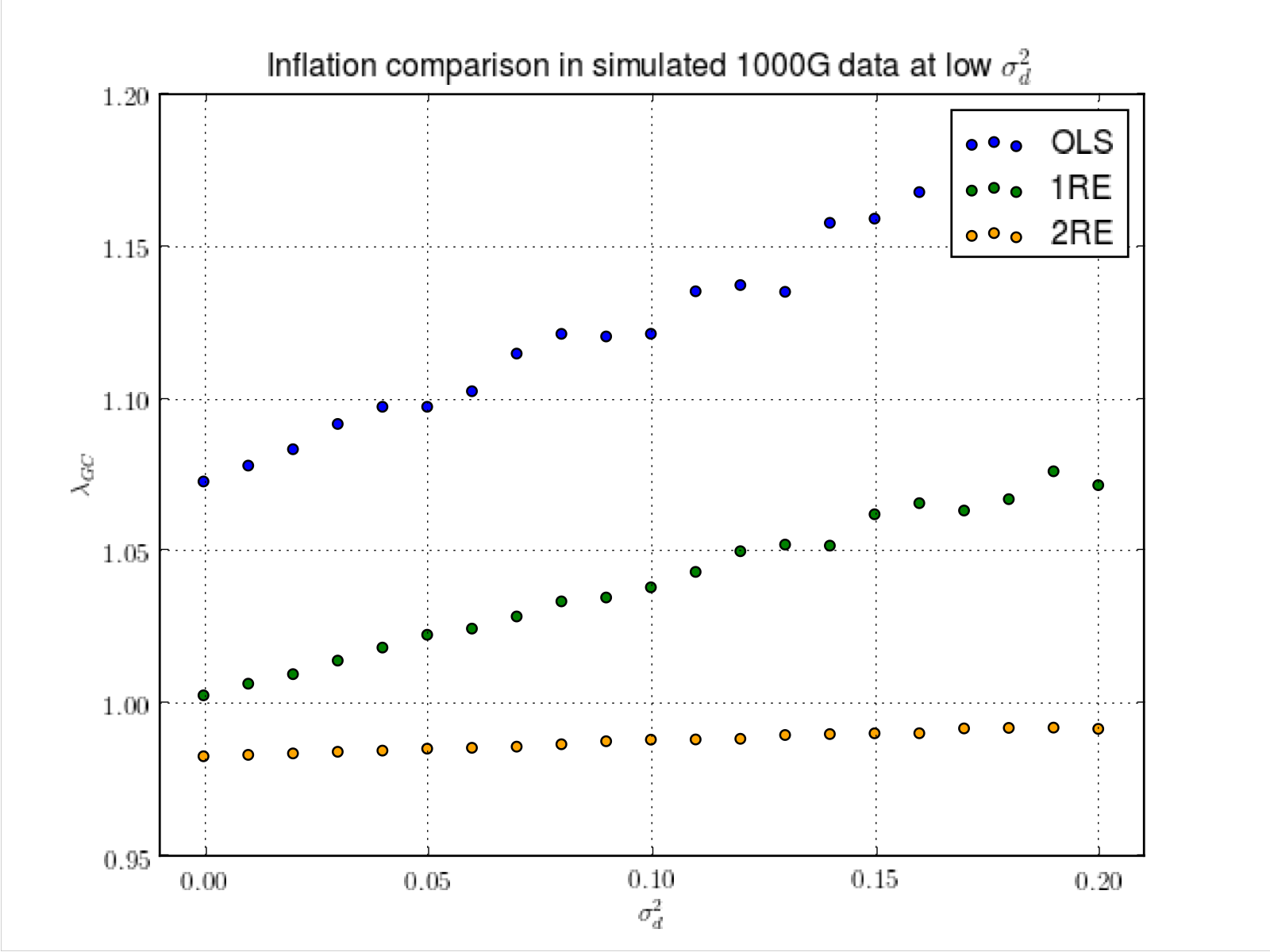

Supplement: S4 Fig — The GxE variance σd2 is taken from 0 to 0.2 while the purely genetic variance σg2 and the purely random variance σe2 are held constant. (TIF) [file pgen.1005849.s004.tif]

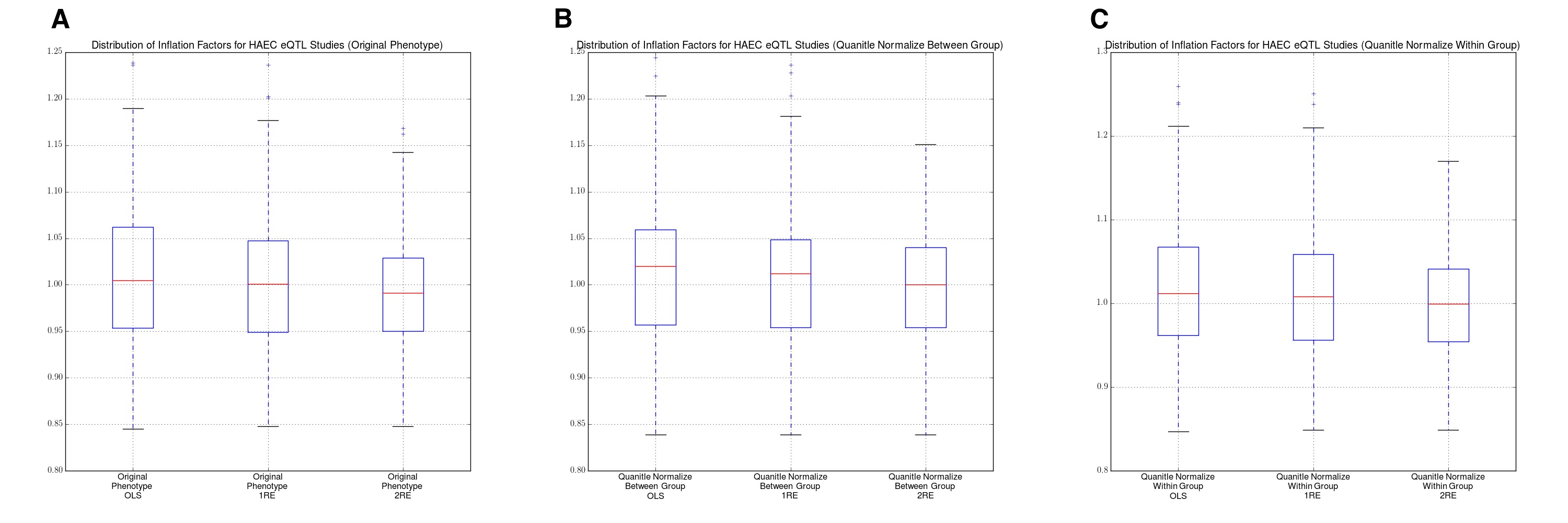

Supplement: S5 Fig — In A, we apply no quantile normalization; in B, the whole sample is first quantile normalized; in C, each environmental group is quantile-normalized separately. There is an apparent increase in performance of OLS and 1RE methods (their λGC becomes closer to 1) using quantile normalization, however, the performance is not as good as 2RE methods. There is little appreciable difference between the population-wide quantile normalization and within-group quantile normalization. (TIFF) [file pgen.1005849.s005.tiff]

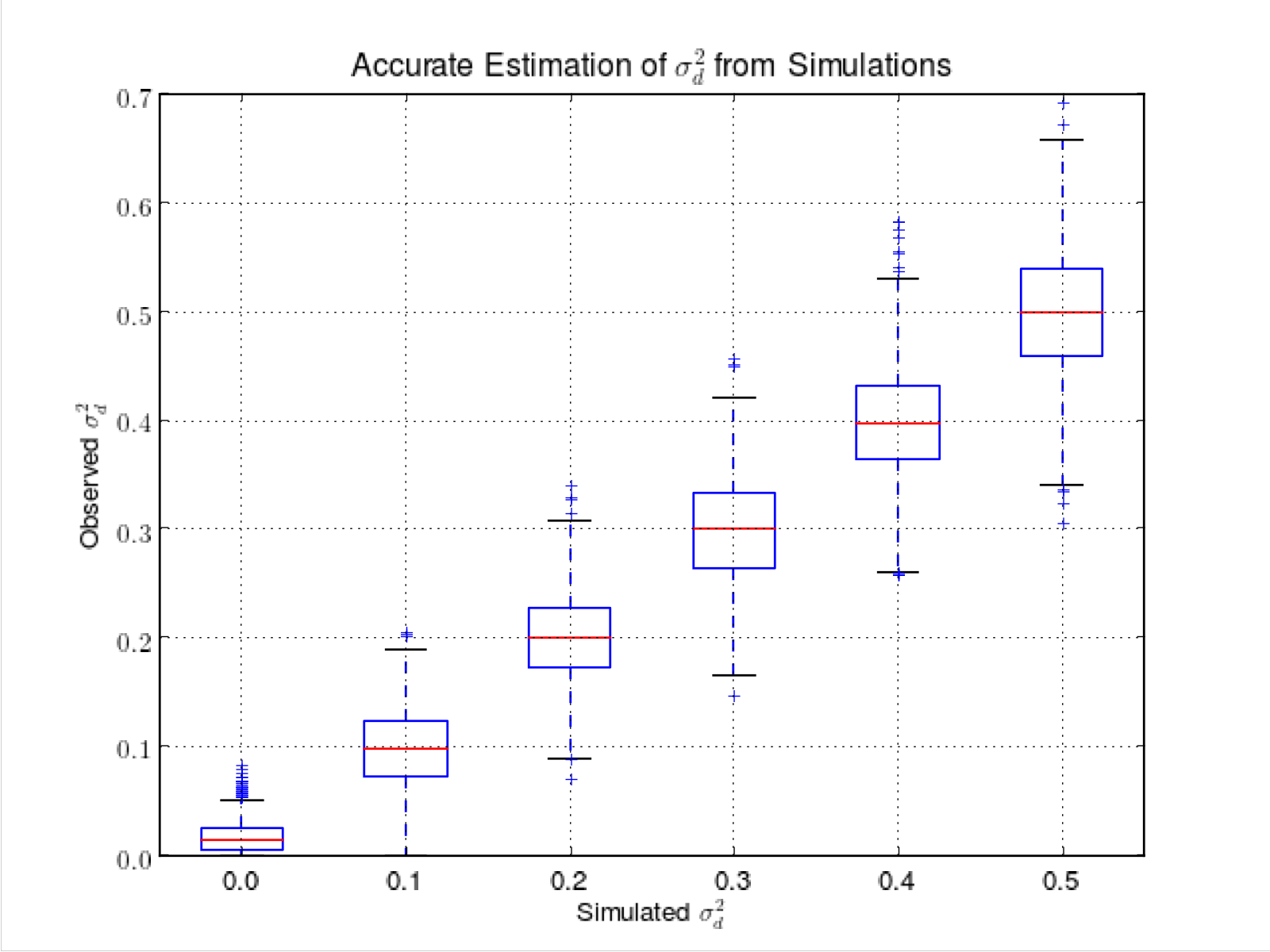

Supplement: S6 Fig — The bias is low, and the variance of the estimate appears to be somewhat proportional to the size of σd2. (TIF) [file pgen.1005849.s006.tif]

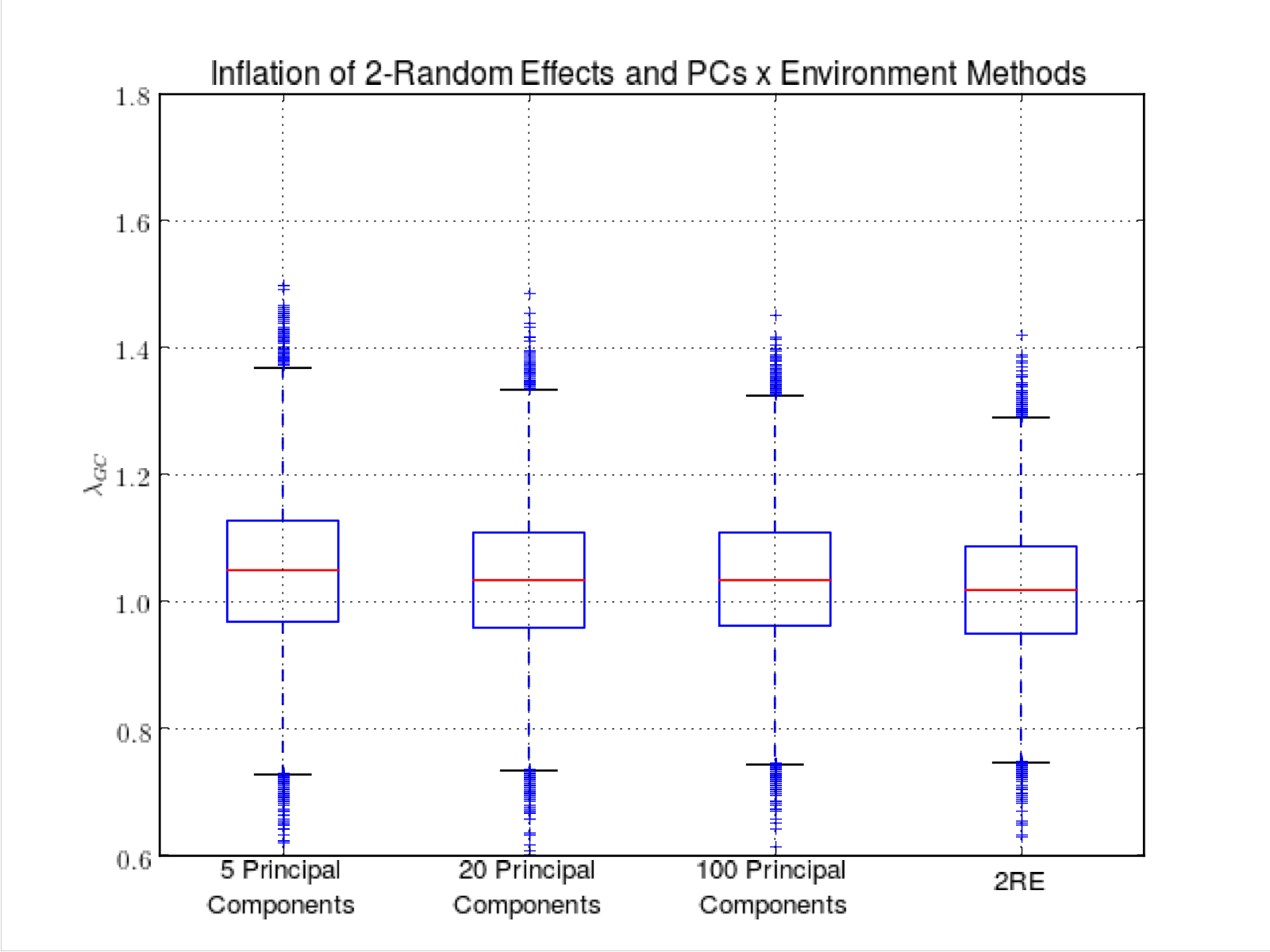

Supplement: S7 Fig — (TIF) [file pgen.1005849.s007.tif]
