# Supplementary material for: Accounting for Population Structure in Gene-by-Environment Interactions in Genome-Wide Association Studies Using Mixed Models
Source: PLoS Genet. 2016 Mar 4;12(3):e1005849. doi: 10.1371/journal.pgen.1005849 (PMC4778803; doi:10.1371/journal.pgen.1005849)
Supplement: S1 Table — The GxE heritability is highly significant for many phenotypes in Table 1, which this table references for the variances explained. GCTA software, using the environmental factor as a covariate, is utilized to estimate the phenotypic variance and its standard error for each phenotype. (PDF) [file pgen.1005849.s008.pdf]

S1 Table: Variance of phenotype explained by the genetic kinship matrix ($\hat{\sigma}_g^2$), when estimated in the absence of a GxE effect. The GxE heritability is highly significant for many phenotypes in Table 1, which this table references for the variances explained. GCTA software, using the environmental factor as a covariate, is utilized to estimate the phenotypic variance and its standard error for each phenotype.

Phenotype	Variance explained by K		Variance explained by K^D		Variance explaiend by K without GxE	
	$\hat{\sigma}_g^2$	SE	$\hat{\sigma}_d^2$	SE	$\hat{\sigma}_g^2$	SE
bw	61.10%	4.55%	8.23%	2.29%	63.8%	4.05%
fat_mass	60.43%	4.93%	13.30%	3.13%	63.8%	3.96%
ffa	41.12%	5.81%	11.30%	3.66%	45.87%	5.78%
ffp	45.00%	6.76%	28.34%	5.58%	56.03%	4.34%
ffp_percentage	41.03%	6.69%	26.90%	5.43%	53.22%	4.45%
free_fluid	36.00%	5.01%	7.68%	2.62%	39.61%	4.48%
gfp	58.07%	5.35%	16.57%	3.75%	64.27%	5.11%
gfp_percentage	58.91%	5.18%	14.86%	3.47%	66.14%	4.76%
glucose_lc	35.82%	5.85%	17.05%	4.17%	44.19%	6.26%
glucose	34.48%	6.16%	19.43%	4.63%	40.97%	5.42%
hdl	60.82%	4.39%	4.51%	1.67%	59.21 %	4.31%
ldl_and_vldl	40.63%	5.02%	6.21%	2.35%	42.58%	4.95%
lean_mass	66.21%	4.16%	7.04%	1.99%	69.51%	4.36%
mfp	48.07%	5.37%	12.05%	3.21%	53.06%	5.58%
mfp_percentage	44.87%	5.39%	11.59%	3.19%	50.32%	5.31%
nmr_bf_percentage	61.60%	4.88%	13.45%	3.13%	67.24%	4.31%
nmr_total_mass	55.82%	5.10%	12.56%	3.08%	62.72%	5.56%
rfp	53.52%	4.79%	5.67%	2.03%	53.89%	4.88%
rfp_percentage	48.75%	4.93%	5.70%	2.12%	49.91%	5.00%
spleen_wt	52.11%	4.93%	5.58%	2.14%	54.81%	4.79%
tc	65.33%	4.05%	3.26%	1.34%	65.90%	4.11%
tg	34.53%	5.72%	15.68%	4.00%	42.89%	6.01%
uc	57.35%	4.99%	7.45%	2.59%	60.28%	4.14%
